# The Prince of Wales' Hospital Stamps

**Published:** 1897-05-22

**Authors:** 


### THE PRINCE OF WALES' HOSPITAL STAMPS.

We paid a visit to Messrs. Simpkin, Marshall, and Co. on the eve of going to press to ascertain how the hospital stamps were taking with the public. We were informed that the demand for them has been so great that the whole of the half-crown stamps which were ready for sale were exhausted on the first day, and that the total day's sales were so large that they far exceeded in number the whole of the issue of the Rowland Hill Memorial Stamps, which it took the General Post Office one week to dispose of. At the present rate it would appear, therefore, that the Prince of Wales' Hospital Stamps will be at an enormous premium long before Commemoration Day, and having this in view we would impress upon the authorities of the Hospital Saturday Fund especially the importance of spreading this information amongst the working classes, or great disappointment is likely to result. The Prince of Wales' primary object in consenting to the issue of these stamps, and especially of the 1s. stamp, was to enable every inhabitant of London to contribute regularly to the hospitals, and to have a pleasant memento of the fact. The stationers and booksellers are publishing tasteful cards, which they are offering for sale with the stamps, so that they may be framed and permanently preserved by the purchasers. We would suggest that some enterprising firm of album makers should issue a small album to take the Prince of Wales' Stamps annually, if this form of collection should be repeated, so that it may be purchased by members of the working classes, who would thus have a tasteful and permanent evidence of the fact that they were regular subscribers to the funds of the hospitals. Apart from these considerations it is highly satisfactory to know that the idea has taken with all classes of the people, and that all who desire to obtain any of the Prince of Wales' Hospital Stamps must order them immediately from the nearest bookseller or stationer, or they will have to pay a high premium for their possession.

